# Urban and Rural Population and Development Research on Medical Coordination: In View of Dalian 2008–2017 Official Statistics

**DOI:** 10.3390/ijerph18126395

**Published:** 2021-06-13

**Authors:** Yukun Qiu, Wei Lu, Jianke Guo, Caizhi Sun, Peng Jia

**Affiliations:** 1 School of Architecture and Arts, Dalian University of Technology, Dalian 116081, China; qiuyukun@mail.dlut.edu.cn; 2 Research Center of Ocean Economy and Sustainable Development, Liaoning Normal University, Dalian 116029, China; guojk2012@lnnu.edu.cn (J.G.); suncaizhi@lnnu.edu.cn (C.S.); 3 School of Resources and Environmental Science, Wuhan University, Wuhan 430079, China; jiapengff@hotmail.com; 4 International Institute of Spatial Lifecourse Epidemiology (ISLE), Hong Kong, China

**Keywords:** public health services, comprehensive development index model, coupling coordination model, urban and rural areas

## Abstract

Providing universal quality health services is one of the Sustainable Development Goals (SDG) to achieve by 2030. We evaluated the sustainable and coordinated development of urban and rural medical care from 2008–2017 in Dalian, China, by developing an evaluation system based on population and health services. We used a comprehensive development index model and a coupling coordination model to evaluate the status and sustainable development of population and medical services in Dalian. The overall level of population development index in urban areas was significantly lower than in rural areas in the past decade. Comparing the data for 2008 and 2017, Zhongshan District (−31.51%), Ganjingzi District (−25.67%), Lyshunkou District (−35.45%), and Pulandian District (−19.59%) posted significant declines in the population development index. The overall medical service development index for both urban and rural areas registered a steady upward trend. In terms of the relationship between population and medical services, a more pronounced coupling running-in stage was observed among urban areas than among rural areas. Among urban areas, the coupling running-in stage in Zhongshan District (2013–2016) and Shahekou District (2011–2014) was most pronounced, while among rural areas, Jinzhou District (2012–2016, 0.684~0.756) had the most distinct coupling running-in stage. In terms of coordination development, we found that both urban and rural areas experienced a long period of moderate coordination stage. Among urban areas, except for some middle and mountainous districts with unstable changes in the coordination degree, the overall development trend in the region showed a stable transition from moderate coordination stage towards high coordination stage. From 2008 to 2017, only the coordination degree in Jinzhou District (−9.17%) showed negative growth. Although considerable efforts have been initiated to improve the coordinated development of Dalian’s urban and rural populations and its medical services, the medical and healthcare systems still face numerous challenges.

## 1. Introduction

In 2000, the goal of providing adequate health care services for all people was included in the United Nation’s Millennium Development Goals (MDGs). In 2015, the target was again included in the Sustainable Development Goals (SDG). In 2018, China scored 78 and ranked 48 in the Lancet Assessment of the Health Quality and Accessibility Index (HAQ) of 195 countries, ranking it as one of the most progressive countries in the middle Socio-Demographic Index [1]. According to the China Health Surveillance Report published by the World Bank and World Health Organization [2], the country’s medical insurance coverage has increased from 28% to 98% since the implementation of medical reforms in 2000. The number of insured people now exceeds 1.35 billion. The rural cooperative medical policy has increased the coverage rate among the rural population, from only 9.5% being covered by the medical cooperation fund to full nationwide coverage. In China, the government has adopted active measures to continue the coordinated development of urban and rural health services [3], including reforming medical systems [4], strengthening universal medical insurance [5], reducing drug prices [6], increasing reimbursable medical expenses [7], and increasing the proportion of new rural cooperative funds [8]. While China has achieved visible results, narrowing the gap between urban and rural medical and health services, achieving the SDG goal of providing universal health services remains a persistent challenge.

After conducting an extensive review of articles published in PubMed from January 2010 to August 2019, we found that, at present, most of the research on the urban and rural medical and health services in China’s large cities has been focused on institutional analysis at the national level [9,10,11] and macro-scale data [12,13,14], while regional evaluation has largely been overlooked. Many of these studies used only single index metrics, such as population [15,16], economy [17,18], and medical resources [19,20], instead of using a comprehensive evaluation system. Thus, their findings often diverge from the actual situation and have limited capacity to provide substantive recommendations to decision-makers. As a relative micro-study, the sustainable and coordinated development of urban and rural populations and medical services in different regions has different existing conditions, which should be considered comprehensively. For this study, we used data provided by the Dalian Municipal Investigation Team of the Dalian Bureau of Statistics, the Bureau of Health, the Bureau of Civil Affairs, and the National Bureau of Statistics. The datasets are essentially different from those provided by the published statistical yearbook. We obtained some hidden details to avoid the impact of missing data on the results of the study.

**Figure 1:**
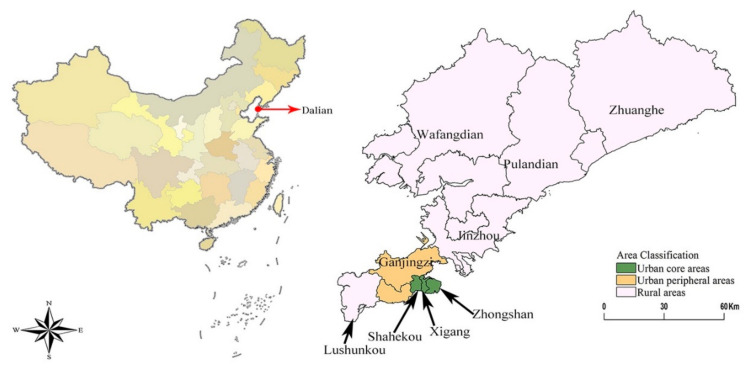
Overview of Dalian City.

To address this current research gap, we focused our study on evaluating the medical services at the city level for both urban and rural communities, focusing mainly on the coordinated development between population development and medical services. For this study, the city of Dalian was selected as the study area (Figure 1). Dalian is one of the 35 representative cities in China chosen by the United Nations Development Agency in its report entitled *China Urban Sustainable Development: Measuring Ecological Investment and Human Development* [21]. The city has shown promising results towards the sustainable and coordinated development of its urban and rural population, particularly in terms of medical and health services. Population and medical and health service data from 2008 to 2017 were obtained from the Dalian City Investigation Team of the National Bureau of Statistics. These data were then used to develop an evaluation system that would assess the level of sustainable and coordinated development in urban and rural medical services. We used the comprehensive development index model to measure the population and medical services for the various regions of Dalian City. We then determined the interaction coupling and coordination model of multiple systems using the capacity coupling coefficient model, which is based on the concept of capacity coupling in physics. This study provides a more realistic assessment of the development of population and medical services, analyzing variations in development for particular areas within a city. The results from this study offer a reasonable scheme to improve health services for a growing population and promote the coordinated development between population and medical services.

## 2. Methods

### 2.1. Study Design and Data Collection

An evaluation system was developed to assess the sustainable and coordinated development in urban and rural health, using demographic data and medical and health service data. Population data were obtained from the Dalian City Investigation Team of the National Bureau of Statistics and the Dalian Statistical Bureau. The City Investigation Team regularly conducts census-related activities to update demographic information. The Bureau of Statistics is responsible for classifying and summarizing the population data collected by the investigation team. Data on health services were acquired from the Bureau of Health and the Bureau of Political Affairs. The Bureau of Health is mainly responsible for collating data on the allocation of medical resources and health service expenditure. Data on health insurance were obtained from the Bureau of Civil Affairs. The data used in this study came from official government records and reports to ensure the data’s quality and accuracy.

Based on Dalian’s population, medical care, and health service data, we developed an evaluation system to analyze the level of development of urban and rural medical services (Table 1). In order to minimize the effects of having indicators with different metrics, we standardized all the indicators before the calculations. We used a comprehensive development index to quantify population and medical and health services and used the analytic hierarchy process (AHP) and the entropy method to calculate the subjective and objective weights for each index. The comprehensive weight model was used to determine the comprehensive weight of each index in the comprehensive development index model. We then used the coordination model to evaluate the coordinated development of the urban and rural population and the medical services in Dalian.

### 2.2. Mathematical Model

#### 2.2.1. Analytic Hierarchy Process (AHP)

We used AHP to obtain the subjective weight for each index. The concept of AHP is based on compartmentalizing a complex multi-objective decision-making problem into multiple targets or criteria as a system and then disaggregating it into several levels of multi-indicators [22,23,24]. The analytic hierarchy process decomposes the problem into different components according to the nature of the problem and the overall goal. The technique combines the factors at different levels according to the interrelationship between the factors and the affiliation relationship, forming a multi-level analysis structure [25]. We used Yaahp software to test the consistency of the index. The test result was 0.0553 (<1), and the results of the index test were statistically significant.

#### 2.2.2. Entropy Weight Method (EWP)

The entropy method was used to obtain the objective weight for each index [26,27,28]. The entropy formula is given as:

(1) $$E_{j}=-\frac{1}{\ln m}\sum_{i=1}^{m}y_{ij}{\ln y}_{ij}$$

(2) $$D_{j}=1-E_{j}$$

(3) $$w_{j}^{\prime \prime }=\frac{D_{j}}{\sum_{j=1}^{n}D_{j}}$$

where $E_{j}$ represents the information entropy of the j-th index in the evaluation matrix *Y*. When the amount of information contained in a particular indicator is consistent with all the research areas, the information entropy of the indicator reaches the maximum value, such that $E_{j}=1$. Before performing entropy weight calculations, the information utility value of the indicator should first be calculated. The utility value $\left(D_{j}\right)$ of the indicator is dependent on the indicator’s information entropy $\left(E_{j}\right)$, which is then used to determine the size of the indicator’s entropy weight $w_{j}^{\prime \prime }$ (Table 2).

#### 2.2.3. Comprehensive Weight Model

In order to overcome the subjectivity of the indicator evaluation by experts and decision-makers in AHP and the entropy weight method, objective evaluation of each index was carried out. The overall weight $w_{j}$ of each index was calculated using the equation:

(4) $$w_{j}=\alpha w_{j}^{\prime }+\left(1-\alpha \right)w_{j}^{\prime \prime }$$

In this study, the value of α was set to 0.5, as recommended by previous research. Using proportional changes in the subjective and objective weights, previous research scholars found that employed sensitivity analysis and found that α equal to 0.5 is relatively reasonable [29,30,31] (Table 3).

#### 2.2.4. Comprehensive Development Index

We divided the indicators into positive and negative indicators [32,33,34]. The specific standardization process is discussed in length. The comprehensive development index formula is:

(5) $$Y_{i}=\sum_{i}^{n}y_{i}^{\prime }\times w_{j}^{\prime \prime },i=1,2$$

where $Y_{1}$ denotes population index; $Y_{2}$ denotes the medical service index, $y_{i}^{\prime }$ is the normalized value of the index in item *i*, and $w_{j}^{\prime \prime }$ is the comprehensive weight of each index, such that $w_{j}^{\prime \prime }$ is the comprehensive weight of each level index. See Table 4 for the comprehensive development index of population and health care in different regions.

#### 2.2.5. Coupling Coordination Model

Referring to the concept of Capacitive Coupling and the capacity coupling coefficient model in physics [35,36], the coupling degree model for multiple systems (or elements) is generalized as follows:

(6) $$C=2\sqrt{\frac{Y_{1}\times Y_{2}}{{\left(Y_{1}+Y_{2}\right)}^{2}}}$$

where C denotes the coupling degree; $Y_{1}$ is population index; and, $Y_{2}$ is the medical service index. In the formula, coupling degree value *C* < [0, 1].

Coordination refers to the different parts of the germplasm for every factor in the evolution process of the system. It is the harmonious and consistent attribute of forming a unified whole. Based on literature, an organic connection exists between population and medical services. Compared with the coupling degree, the coupling coordination model can better reflect the degree of coordination between population and medical services and is given by the following equations:

(7) $$Y=\alpha Y_{1}+\beta Y_{2}$$

(8) $$D=\sqrt{C\times Y}$$

where *D* is the coupling coordination degree; *C* is the coupling degree, *Y* is the comprehensive evaluation index of population and health services, and *α* and *β* are the undetermined weights. As population and health services are equally important, *α* and *β* are both given the value of 0.5. See Table 5 for the specific division criteria of coupling degree and coordination degree, and Table 6 for the coupling degree and coordination degree of different regions.

### 2.3. Statistical Analysis

We used the comprehensive development index model and the coupling coordination degree model to calculate the comprehensive development indices for population and the medical and health services, the coupling degree, and the coupling coordination degree of urban and rural areas. We used Origin 2018 to draw the population development index and medical service development index, and the contour change map of the coupling degree and coordination degree for 2008–2017. We used ArcGIS10·5 software to draw the comprehensive development indices and the coupling degree in Dalian for 2008, 2012, and 2017.

## 3. Results

### 3.1. The Difference of Population and Medical Comprehensive Development Index in Urban and Rural Areas

**Figure 2:**
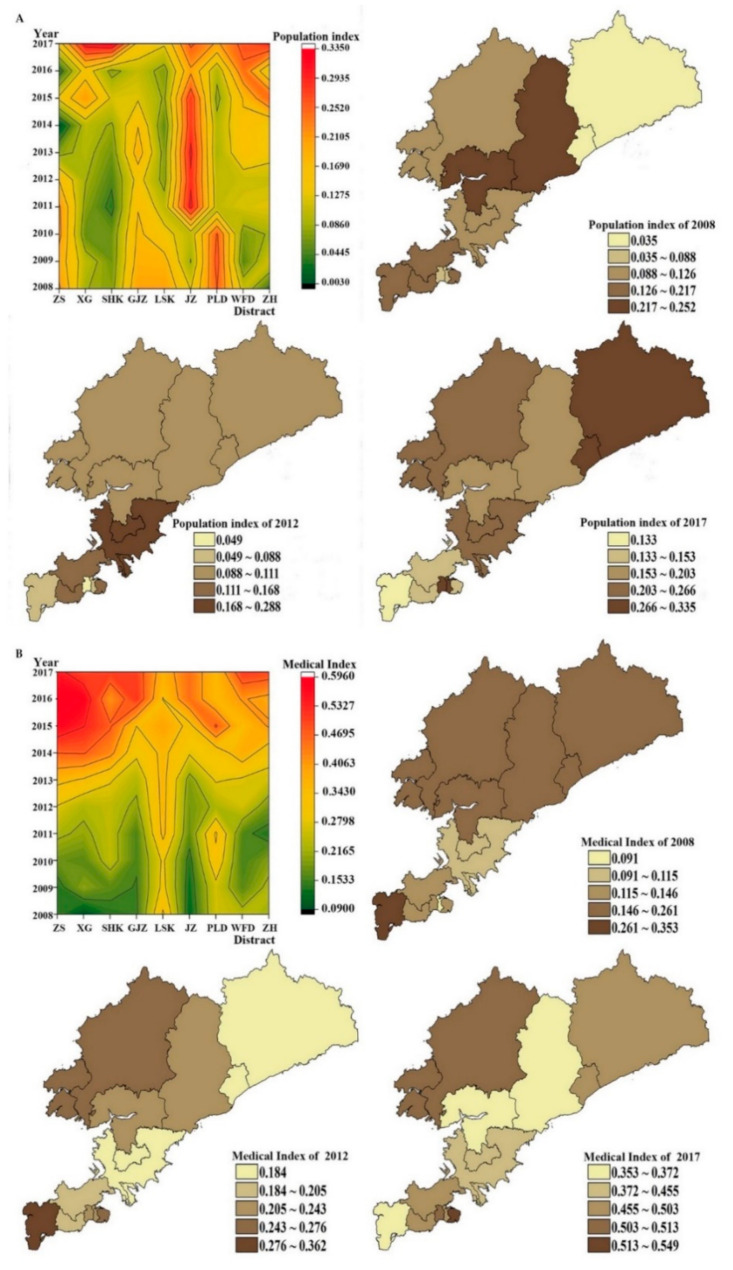
Population index and medical index between urban and rural areas (**A** for Population index, **B** for medical index).

Changes in the population development index between urban and rural areas in Dalian were significant from 2008~2017 (Figure 2A). The overall population development index in urban areas was low. The population development index in Zhongshan District was the most unstable, fluctuating from 0.075 to 0.003 for 2013–2014 and from 0.003 to 0.137 for 2014–2015. Xigang and Shahekou Districts experienced long-term low-level development from 2008 to 2014. The overall level of population development index among rural areas was relatively high. Except for Lushunkou District, other rural areas experienced a long-term high-level development trend. For instance, Jinzhou District experienced a very significant high-level development trend in 2011–2015, growing from 0.280 to 0.304.

The change in population development index from 2008 to 2017 was then analyzed for each region (Figure 2A). We found that the population development index declined considerably for Zhongshan (−31.51%), Ganjingzi (−25.67%), Lushunkou (−35.45%), and Pulandian (−19.59%) Districts. Among urban areas, the largest increase was in Shahekou District (0.073–0.335, 356.10%), while among rural areas, Zhuanghe City (0.035–0.297, 752.94%) registered the highest growth. 

Compared with the population development index, the overall development index in medical services among urban and rural areas showed a steady upward trend (Figure 2B). After 2014, the development index of medical services in urban areas increased significantly, with the highest values found in Zhongshan (0.594) and Xigang Districts (0.579) in 2016. Compared with urban areas, the improvements in the development index of medical services among rural areas were not significant. Jinzhou was the only district to achieve a stable growth trend. By comparing the regional medical service development index (Figure 2B) in 2008 and 2017, we found that both urban and rural areas had positive growth in the medical service development index. The medical service development index in urban areas increased by more than 200%, with Xigang District (0.091~0.495, 446.72%) experiencing the largest increase at 446.72% (0.091–0.495). Among rural areas, the largest increase was in Jinzhou District (0.115–0.455, 294.30%)

We calculated the difference between the comprehensive index of population status and the medical and health service index, and the results are in Table 7. As shown in Table 6, the difference between the comprehensive index of population status in urban areas and the medical and health services index was not significant from 2008 to 2012. Among rural areas, Lushun Port District (2011 0.255; In 2012, 0.288) and Pulandian (2011, 0.246) had large differences. From 2013 to 2017, the difference between the comprehensive index of population status and the medical and health services index in urban and rural areas increased significantly. Significant difference changes were observed in Zhongshan District (2016 0.569), Xigang District (2016 0.430), Shahekou District (2015 0.356), and Ganjingzi District (2016 0.408). Although the difference in rural areas showed significant changes in 2015–2017, the change was relatively small compared to urban areas. By 2017, Xigang district (0.181), Shahekou District (0.173), Jinzhou (0.194), PuLanDian area (0.150) in the population present situation and better coordination of medical and health services. and in (0.401), was seated (0.350), Lyshunkou (0.239), Wafangdian city (0.247), Zhuanghe (0.204) of the population present situation and the medical and health services optimization has the larger development space.

### 3.2. The Difference of Coupling Degree between Population and Medical Service in Urban and Rural Areas

**Figure 3:**
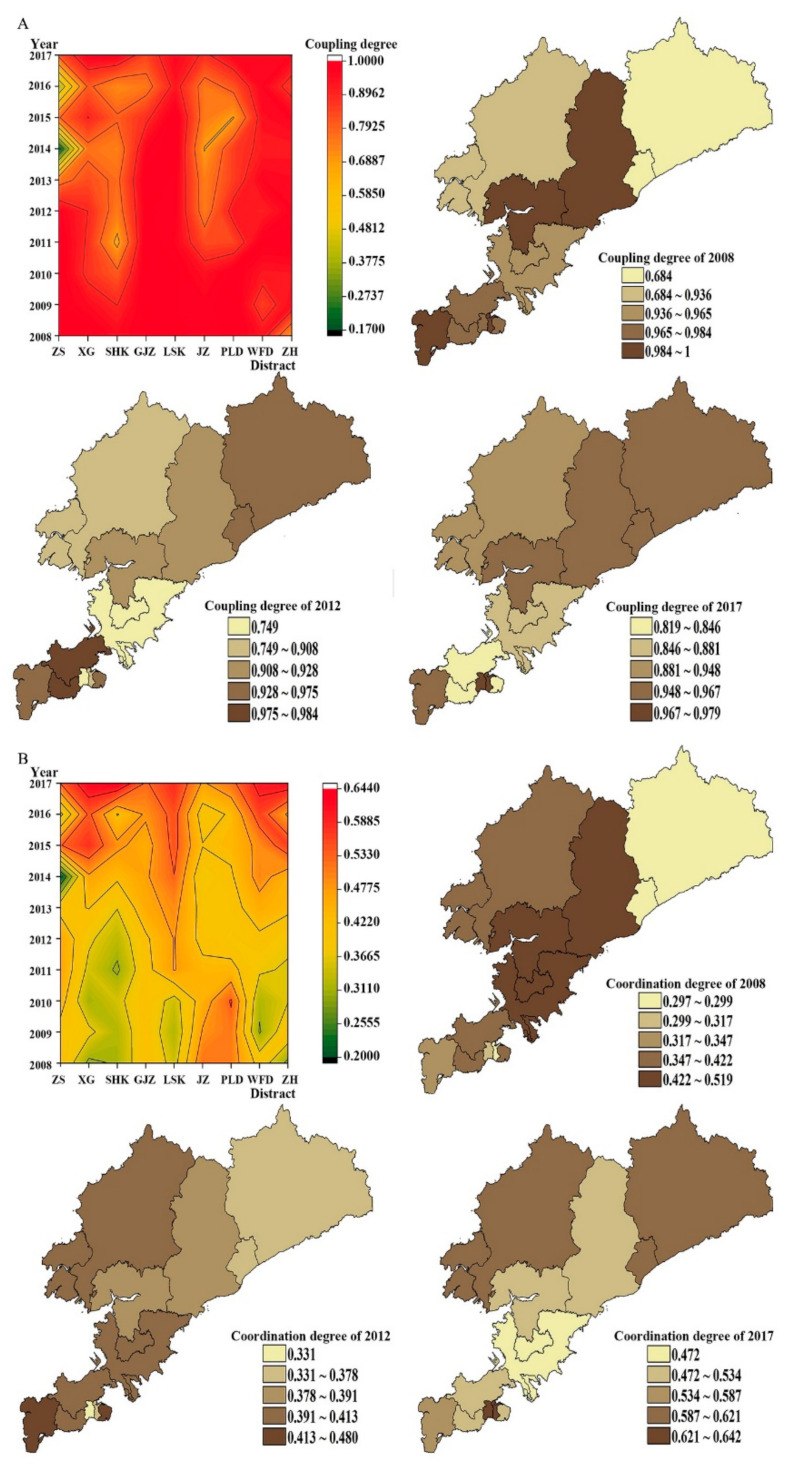
Change trend of coupling degree and coordination degree between urban and rural areas.

In 2008–2017, there was a noticeable coupling run-in stage between urban areas and rural areas (Figure 3A). The coupling degree between population and medical services in urban areas was unstable, and there was a relatively long coupling run-in stage in Zhongshan District (2013–2016) and Shahekou District (2011–2014), and obvious low-level coupling occurred in Zhongshan District (0.171) in 2014. Among rural areas, Lyshunkou District (0.962–1) and Wafangdian City (0.852–0.988) maintained high levels of coupling, while Jinzhou District (2012–2016, 0.684–0.756) experienced a long period of coupling and running-in. Comparing the coupling degree between population and medical services, we found that most areas in Dalian City experienced a decrease in the coupling for 2008–2017. The few areas that experienced positive growth include Shahekou District (2.66%), Wafangdian City (1.34%), and Zhuanghe City (41.39%).

### 3.3. The Difference in Coordination between Population and Medical Service in Urban and Rural Areas

**Figure 4:**
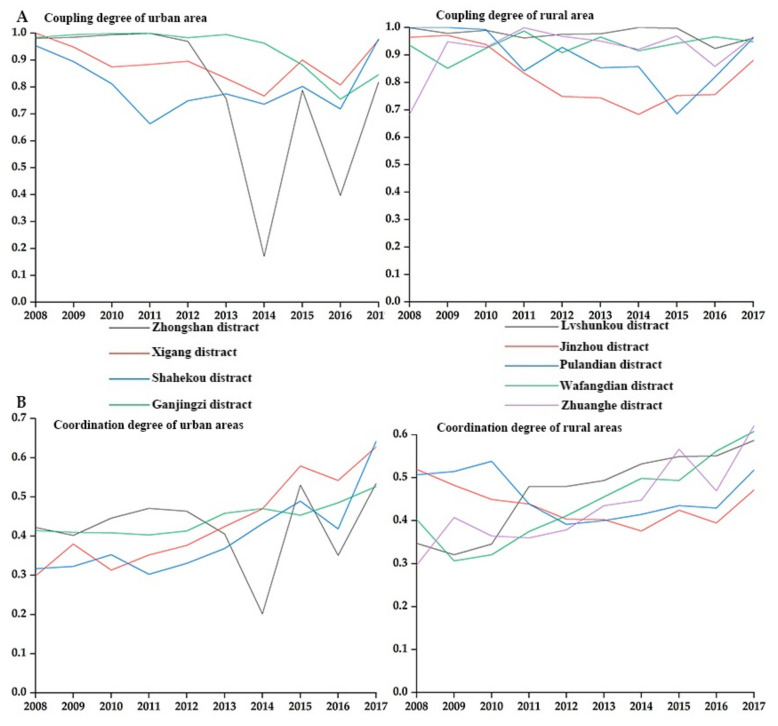
Change in coupling and coordination between urban and rural areas.

Note that the decline in the coupling degree is significantly higher among urban areas than rural areas. Among urban districts, the highest negative growths were found in Zhongshan District and Ganjingzi District at more than 10%, while among rural areas, the highest rate was in Jinzhou District at 8.71%. We also found that both urban and rural areas experienced long moderate coordination (Figure 4A). Except for Zhongshan District, with an unstable movement in coordination degree, most urban areas had a generally stable transition from the moderate coordination stage towards the high coordination stage. The overall coordination degree in Lushunkou District and Wafangdian City indicates a steady growth trend in rural areas. Lushunkou District (2008–2013) achieved a high level of coordinated development for population and medical services in only six years, significantly less than their urban counterparts. We also found that only Jinzhou District (−9.17%) showed negative growth in the coordination degree for population and medical services from 2008 to 2017 (Figure 4B). The highest growth rate among rural areas reached 109.31%, much higher than in urban areas at 83.66%.

## 4. Discussion

From 2008 to 2017, Dalian’s overall development in medical security system was relatively good, but there were still significant differences in the development between urban and rural areas. We analyzed the index weight in the evaluation system. We found that the parameters population over 60 years old, the number of employees insured, and the number of medical institutions belongs to the top five indicators for both urban and rural areas. Among urban areas, the parameters, population over 60 years old (C2, 10.04–15.22%) and the number of medical institutions (C11, 11.67–13.51%), were found to have very strong effects on the sustainable and coordinated development of medical care. In comparison, the parameters, number of medical institutions (C11, 10.17–12.96%), and the government medical expenditure (C12, 10.29–13.87%), were found to significantly affect the sustainable and coordinated development of medical care among rural areas.

Pragmatically resolving the growing medical needs of an increasingly aging population has become a major issue in many urban areas, particularly ensuring a sustainable and coordinated development in medical care and health services. For rural areas, we found that sustainable and coordinated development in medical care depends mainly on the number of medical institutions and government expenditure. In some areas, the contributions from government medical expenditure were as high as 13.87% (Pulandian) and 13.21% (Wafangdian). Significant private investments towards local health services must be encouraged to minimize the over-reliance on government support, particularly among rural communities.

Our results also showed that the development trend in medical services has been more stable than the population index. This suggests that while Dalian was able to improve its medical and healthcare sector, such improvements did not directly translate into raising the population development level. When we calculated the difference between the population and the medical service development indexes for each region, except for Wafangdian, most areas had a decreasing trend. When we looked further into the trends, we found that parts of the city had varying levels of coordination. For instance, the difference between population and medical service development indexes in Xigang District (urban) was 64.87 times higher than in Jinzhou District (rural). In general, the index differences were considerably higher in urban areas compared to their rural counterparts. Such disparities between urban and rural areas may seriously affect the overall coordination in population and medical care development and hinder meaningful progress to the city’s healthcare system. Dalian’s urban areas should seriously consider adopting major changes in policy and strategies that would focus on improving coordination development of the population and medical services.

Our results also show that although the city has a high overall coupling degree, Dalian has not reached a high degree of coordination. Lyshunkou District was the only area to register continued growth in coordination degree. In 2017, most areas reached high coordination in population and medical service development. We then evaluated the population development index, medical service development index and coordinated dispatch for the different regions. Our results show that with continued improvements in medical services, population development became a major determinant influencing the coordination of urban and rural medical care in Dalian. Measures and policies aimed at improving the coordinated development of population and medical services should not only focus on improving the medical and healthcare system, but also include reducing the impact of population development on the sustainable and coordinated development of urban and rural medical care.

## 5. Conclusions

The results of this study have some useful applications to multiple stakeholders, including policymakers. Compared with previous studies, we deliberately chose to use a city-level perspective to explore and assess the improvements in the public health system and its impact on the health of a city. The results of this study have some useful applications to multiple stakeholders, including policymakers. Compared with previous studies, we deliberately chose to use a city-level perspective to explore and assess the improvements in the public health system and its impact on the health of a city. By combining the comprehensive development index model and the coupling coordination model, we were able to analyze changes to the population and medical services for different parts of the city and evaluate the level of coordination in development in terms of coupling degree and coordination degree.

Over the past decade, Dalian has made significant efforts to improve its medical and healthcare services. However, if the city is unable to achieve a high level of coordination in the development of its medical services and its urban and rural populations, the impact of improvements to the healthcare system would be significantly reduced. The UN Sustainable Development Goals on healthcare would then be difficult to achieve. Reaching sustainable and coordinated development of urban and rural medical care in Dalian remains highly problematic. The city’s aging population, the number of medical institutions, and government medical expenditures have become the primary factors hindering development. More attention should be given to improving the coordination degree in urban parts of the city compared with rural areas. More effective policies on population and medical services should be implemented in various regions to promote the sustainable and coordinated development of urban and rural medical care.

## 6. Limitations

There were a number of limitations in this study that could be improved in future research. First, due to constraints in data availability, we included only a limited number of indicators in our proposed evaluation system. Future studies can incorporate other healthcare indicators, such as bed utilization rate and hospital consultation, to improve the assessment. Second, the proposed evaluation index system is composed mainly of objective data. Subsequent research may look into including subjective data, such as resident satisfaction with medical services and infrastructure obtained through questionnaire surveys, to provide a more comprehensive assessment of the healthcare system.

## Figures and Tables

**Table 1:** The evaluation system for the sustainable and coordinated development of urban and rural medical care.

Primary Index	Secondary Index	Tertiary Index	Average Value	Positive/Negative Indicator Direction
Demographic factors (A1)	Population composition (B1)	Population size (C1)	5,903,700 people	−
		Population over 60 (C2)	1.209 million people	−
		Rural population (C3)	2.109 million people	−
Medical service factors (A2)	Medical insurance (B2)	Number of residents participating in medical insurance (C4)	75,400 people	+
		The proportion of residents participating in medical insurance (C5)	16.81%	+
		Number of employees participating in medical insurance (C6)	3.055 million people	+
		The proportion of employees participating in medical insurance (C7)	66.62%	+
	Medical resource allocation (B3)	Number of doctors per thousand (C8)	2.94 people	+
		Number of nurses per thousand (C9)	3.23 people	+
		Number of beds per thousand people (C10)	6.08 beds	+
		Number of medical institutions (C11)	123 institutions	+
		Government medical expenditure (C12)	1886.638 million yuan	+
		The proportion of medical expenditure in total public utility expenditure (C13)	5.39%	+

**Table 2:** AHP and EWP values in urban and rural areas.

	AHP	EWP								
Indicators		City (%)				Rural (%)				
		ZS	XG	SHK	GJZ	LSK	JZ	PLD	WFD	ZH
C1	6.55	8.22	6.75	6.29	4.94	10.50	4.64	4.18	11.28	3.30
C2	6.76	12.36	16.23	20.22	4.89	5.91	2.50	3.29	10.93	7.92
C3	6.90	0.00	0.00	0.00	9.04	7.10	4.18	6.30	7.29	8.14
C4	7.53	7.21	7.59	9.14	6.45	6.97	5.09	6.89	6.44	6.36
C5	6.87	3.67	3.85	3.77	3.73	6.23	6.11	4.49	10.55	10.00
C6	8.22	7.48	7.97	5.49	4.93	3.54	11.02	5.59	5.62	7.88
C7	6.57	3.25	3.09	3.18	8.84	7.81	3.99	6.53	3.35	8.83
C8	6.02	6.32	7.60	4.03	14.79	5.73	6.62	2.70	6.91	6.33
C9	6.61	6.83	6.83	6.68	5.58	6.52	4.78	5.67	5.54	6.81
C10	6.26	8.08	7.05	6.39	8.23	6.96	11.94	4.67	6.04	8.44
C11	10.65	9.76	14.27	13.51	2.30	3.25	4.26	19.67	4.96	8.65
C12	10.43	11.53	5.99	7.99	11.67	5.67	10.68	5.87	13.21	6.29
C13	10.63	7.92	6.07	5.90	9.68	13.73	19.63	9.31	5.62	7.51

ZS—Zhongshan district; XG—Xigang district; SHK—Shahekou district; GJZ—Ganjingzi district; JZ—Jinzhou district; LSK—Lyushunkou district; PLD—Pulandian district; WFD—Wafangdian district; ZH—Zhuanghe district.

**Table 3:** Comprehensive weight of each region.

Indicators	City (%)				Rural (%)					
	ZS	XG	SHK	GJZ	LSK	JZ	PLD	WFD	ZH	AHP
C1	8.22	8.75	8.29	4.94	4.64	6.50	6.18	6.28	6.37	6.55
C2	14.36	14.23	15.22	4.89	4.50	5.91	6.29	6.93	7.92	6.76
C3	-	-	-	9.04	6.18	7.10	6.30	6.29	8.14	6.90
C4	7.21	7.59	7.14	7.45	6.09	6.97	6.89	6.44	6.36	7.53
C5	4.67	4.85	4.77	4.73	6.11	6.23	6.49	6.55	7.12	6.87
C6	9.48	9.97	9.49	4.93	9.02	9.54	8.59	9.62	8.88	8.22
C7	5.25	5.09	5.31	8.84	5.99	7.81	6.53	6.35	7.83	6.57
C8	8.32	8.60	8.54	14.79	6.62	5.73	6.89	6.91	6.33	6.02
C9	6.83	6.83	6.68	6.58	6.78	6.68	5.67	5.54	6.16	6.61
C10	8.45	9.34	8.39	8.23	9.50	6.96	6.32	6.04	6.44	6.26
C11	12.76	12.27	13.51	4.23	4.26	5.17	10.67	10.96	10.65	10.65
C12	6.53	5.99	5.87	11.67	10.68	11.67	13.87	13.21	10.29	10.43
C13	7.9%	6.49	6.79	9.68	19.63	13.73	9.31	8.88	7.51	10.63

ZS—Zhongshan district; XG—Xigang district; SHK—Shahekou district; GJZ—Ganjingzi district; JZ—Jinzhou district; LSK—Lyushunkou district; PLD—Pulandian district; WFD—Wafangdian district; ZH—Zhuanghe district.

**Table 4:** The comprehensive development index of population and health care in different regions.

Year	Y1 (Population Index) Y2 (Medical Service Index)	City				Rural				
		ZS	XG	SHK	GJZ	LSK	JZ	PLD	WFD	ZH
2008	y1	0.217	0.088	0.073	0.206	0.206	0.126	0.253	0.112	0.035
	y2	0.146	0.091	0.137	0.143	0.353	0.115	0.261	0.235	0.223
2009	y1	0.192	0.104	0.065	0.187	0.183	0.084	0.266	0.052	0.120
	y2	0.136	0.200	0.169	0.150	0.296	0.127	0.263	0.168	0.231
2010	y1	0.222	0.058	0.064	0.177	0.141	0.104	0.256	0.069	0.090
	y2	0.178	0.166	0.243	0.157	0.289	0.137	0.327	0.154	0.196
2011	y1	0.216	0.075	0.035	0.167	0.103	0.304	0.105	0.120	0.124
	y2	0.227	0.206	0.241	0.158	0.358	0.174	0.351	0.165	0.135
2012	y1	0.168	0.088	0.049	0.142	0.073	0.288	0.103	0.108	0.111
	y2	0.276	0.228	0.243	0.205	0.362	0.184	0.227	0.264	0.185
2013	y1	0.075	0.096	0.064	0.190	0.072	0.302	0.090	0.159	0.138
	y2	0.359	0.336	0.286	0.232	0.363	0.196	0.286	0.271	0.260
2014	y1	0.003	0.103	0.082	0.168	0.056	0.280	0.097	0.162	0.133
	y2	0.471	0.473	0.425	0.292	0.358	0.286	0.304	0.381	0.303
2015	y1	0.137	0.210	0.120	0.123	0.082	0.280	0.075	0.172	0.250
	y2	0.576	0.535	0.477	0.343	0.397	0.324	0.478	0.345	0.412
2016	y1	0.025	0.149	0.074	0.107	0.071	0.203	0.096	0.242	0.125
	y2	0.594	0.579	0.413	0.516	0.341	0.454	0.354	0.411	0.388
2017	y1	0.148	0.314	0.335	0.153	0.133	0.260	0.203	0.266	0.297
	y2	0.549	0.495	0.508	0.503	0.372	0.455	0.353	0.513	0.501

ZS—Zhongshan district; XG—Xigang district; SHK—Shahekou district; GJZ—Ganjingzi district; JZ—Jinzhou district; LSK—Lyushunkou district; PLD—Pulandian district; WFD—Wafangdian district; ZH—Zhuanghe district.

**Table 5:** Value range and corresponding explanation of coupling degree and coordination degree.

	C (Coupling Degree)	D (Coupling Coordination Degree)
(0, 0.3]	The system is at a lower level of coupling.	Low-coordinated coupling
(0.3, 0.5]	The coupling of the system is in a period of stagnation.	Moderately coordinated coupling
(0.5, 0.8]	The coupling of the system enters the running-in stage, and the two become benign coupling.	Highly coordinated coupling
(0.8, 1.0]	The system is at a high level of coupling.	Extremely coordinated coupling
1	The coupling degree of the system is the largest, and the system achieves a benign resonance coupling and tends to a new ordered structure.	Extremely coordinated coupling

**Table 6:** For the coupling degree and coordination degree of different regions.

Year	C (Coupling Degree) D (Coupling Coordination Degree)	City				Rural				
		ZS	XG	SHK	GJZ	LSK	JZ	PLD	WFD	ZH
2008	C	0.981	1.000	0.953	0.984	0.999	0.965	1.000	0.936	0.684
	D	0.422	0.299	0.317	0.414	0.347	0.519	0.507	0.403	0.297
2009	C	0.985	0.948	0.894	0.994	0.979	0.972	1.000	0.852	0.949
	D	0.402	0.380	0.323	0.410	0.321	0.482	0.514	0.306	0.408
2010	C	0.994	0.875	0.812	0.998	0.991	0.938	0.993	0.925	0.928
	D	0.446	0.313	0.353	0.408	0.346	0.449	0.538	0.321	0.364
2011	C	1.000	0.884	0.663	1.000	0.962	0.834	0.842	0.988	0.999
	D	0.471	0.352	0.303	0.403	0.480	0.439	0.439	0.375	0.360
2012	C	0.970	0.896	0.749	0.984	0.975	0.749	0.928	0.908	0.968
	D	0.464	0.376	0.331	0.413	0.480	0.404	0.391	0.411	0.378
2013	C	0.756	0.833	0.774	0.995	0.977	0.744	0.853	0.966	0.951
	D	0.405	0.424	0.368	0.458	0.493	0.402	0.400	0.456	0.435
2014	C	0.171	0.767	0.736	0.963	1.000	0.684	0.858	0.915	0.921
	D	0.201	0.470	0.432	0.470	0.532	0.376	0.415	0.498	0.448
2015	C	0.788	0.900	0.803	0.882	0.997	0.753	0.685	0.943	0.969
	D	0.530	0.579	0.489	0.454	0.549	0.424	0.435	0.494	0.566
2016	C	0.397	0.807	0.719	0.755	0.924	0.756	0.820	0.966	0.859
	D	0.351	0.542	0.418	0.485	0.551	0.395	0.430	0.562	0.469
2017	C	0.819	0.975	0.979	0.846	0.962	0.881	0.963	0.948	0.967
	D	0.534	0.628	0.642	0.527	0.587	0.472	0.518	0.608	0.621

ZS—Zhongshan district; XG—Xigang district; SHK—Shahekou district; GJZ—Ganjingzi district; JZ—Jinzhou district; LSK—Lyushunkou district; PLD—Pulandian district; WFD—Wafangdian district; ZH—Zhuanghe district.

**Table 7:** Difference between population index and medical and health service index in urban and rural areas (Y1–Y2).

	City				Rural				
Year	ZS	XG	SHK	GJZ	LSK	JZ	PLD	WFD	ZH
**2008**	0.070	−0.003	−0.064	0.063	−0.147	0.011	−0.008	−0.122	−0.188
**2009**	0.056	−0.097	−0.104	0.036	−0.113	−0.043	0.004	−0.115	−0.111
**2010**	0.043	−0.109	−0.179	0.020	−0.148	−0.033	−0.071	−0.085	−0.107
**2011**	−0.010	−0.131	−0.207	0.009	−0.255	0.130	−0.246	−0.045	−0.011
**2012**	−0.108	−0.141	−0.193	−0.062	−0.288	0.104	−0.123	−0.156	−0.074
**2013**	−0.284	−0.240	−0.222	−0.042	−0.291	0.105	−0.196	−0.111	−0.123
**2014**	−0.467	−0.370	−0.343	−0.124	−0.302	−0.006	−0.206	−0.219	−0.170
**2015**	−0.439	−0.325	−0.356	−0.220	−0.315	−0.044	−0.403	−0.173	−0.163
**2016**	−0.569	−0.430	−0.339	−0.408	−0.270	−0.251	−0.258	−0.169	−0.263
**2017**	−0.401	−0.181	−0.173	−0.350	−0.239	−0.194	−0.150	−0.247	−0.204

## Data Availability

Our data comes from https://stats.dl.gov.cn/ (accessed on 10 June 2021).
